# A Comprehensive Review on Biology, Genetic Improvement, Agro and Process Technology of German Chamomile (*Matricaria chamomilla* L.)

**DOI:** 10.3390/plants11010029

**Published:** 2021-12-23

**Authors:** Ramesh Chauhan, Sanatsujat Singh, Vikas Kumar, Ashok Kumar, Amit Kumari, Shalika Rathore, Rakesh Kumar, Satbeer Singh

**Affiliations:** 1 Division of Agrotechnology, Council of Scientific and Industrial Research—Institute of Himalayan Bioresource Technology (CSIR-IHBT), Palampur 176061, India; ramesh@ihbt.res.in (R.C.); sanatsujat@ihbt.res.in (S.S.); ashok@ihbt.res.in (A.K.); rakeshkumar@ihbt.res.in (R.K.); 2 Division of Environmental Technology, CSIR-IHBT, Palampur 176061, India; vikas@ihbt.res.in; 3 Division of Chemical Technology, CSIR-IHBT, Palampur 176061, India; amitkumari@ihbt.res.in; 4 Academy of Scientific and Industrial Research, Ghaziabad 201002, India; shalikarathore13@gmail.com

**Keywords:** chamomile, pharmacological uses, genetic improvement, agronomy, essential oil

## Abstract

German chamomile (*M. chamomilla*) is recognized as a star herb due to its medicinal and aromatic properties. This plant is found across a wide range of climatic and soil conditions. Both the flower heads and blue essential oils of German chamomile possess several pharmacological properties of an anti-inflammatory, antimicrobial, antiseptic, antispasmodic and sedative, etc., nature, which makes it a highly sought after herb for use in many pharma and aroma industries. Chamomile tea, prepared from its flower heads, is also a well-known herbal tea for mind and body relaxation. Though it is a high-demand herb, farmers have not adopted this plant for large scale cultivation as a crop, which could improve their livelihood, due to the high cost in flower heads harvesting, loss in over mature and immature flower heads picking during harvesting, unavailability of varieties and agrotechnologies for machine harvesting, a lack of efficient process development of oil extraction and in the lack of improved stable varieties. There are many studies that have reported on the phytochemistry and pharmacological uses of chamomile, which further explore its importance in the medicine industry. Several studies are also present in the literature on its cultivation practices and plant ecology. However, studies on breeding behavior, genetic improvement, varietal development and mechanical harvesting are scarce in German chamomile. Hence, keeping in mind various aspects of farmers’ and researchers’ interest, earlier reports on taxonomy, floral biology, processing of oil extraction, active constituents, uses, agronomy, breeding challenges and opportunities in German chamomile are summarized in this review.

## 1. Introduction

German chamomile (*M. chamomilla*) is an annual medicinal and aromatic herb which is found in south east Europe and in adjoining Asian countries. It has a wide adaptability over range of soil type, cold and soil alkalinity. German chamomile has been considered as a medicinal treasure due to its extensive therapeutic use. Hence, making it the fascinating herb of the earth. It has been used as a medicinal plant since the classical period, and the Egyptians considered this herb a sacred gift by the God ‘Sun’ [1]. Due to having several aromatic and pharmacological properties, it is popularly known as a “star” herb [2].

German chamomile flower heads have been used as such in herbal tea or in various forms of preparation from the extracts. The flower heads, and their extracts of German chamomile, are used in several herbal remedies, herbal tea, cosmetics, food flavors, dye and pest repellent [3,4]. The essential oil of German chamomile is of high pharmaceutical value and contains flavones, polysaccharides and lipophilic active ingredients, corresponding to German chamomile activity. The primary use of essential oils is in the food industry, aromatherapy industries and perfumery; due properties of anti-inflammatory, antiulcerogenic, antimicrobial, antiseptic, antispasmodic, sedative, immunomodulatory and wound healing [5], its oil is also vital in the pharmaceutical industry.

Despites its great economic value and large demand, this herb is not popular among farmers at a commercial level, due to a lack of available varieties, a lack of appropriate agrotechnologies, the high cost of harvesting and challenges of losing active constituents in the process of oil extraction. In assessing the quality of German chamomile and its extracts, lipophilic active ingredients are of enormous importance. In addition to (−)-α -bisabolol and chamazulene, poly-ynes such as spiroethers are also important. Spiroethers have anti-inflammatory and spasmolytic properties but are easily decomposed, especially at slightly elevated temperatures. Hence, the extraction process of bio-active constituents is crucial. The German chamomile has some varieties of both a diploid (2 n = 18) and tetraploid (2 n = 36) genetic load. The diploid varieties were reported to be shorter in growth and height than the tetraploid varieties [6]. The plant lacks synchronous flowering, which cause difficulties in mechanical harvesting and leads to a high cost of labor for flower heads picking. So, this crop requires extensive research and development in the areas of agrotechnology developments, genetic improvement, varietal development and in the processing of oil extraction for its large scale cultivation and industrial applications, which necessitates an assessment of the important parameters for extraction of active constituents.

Thus, the present review was undertaken to understand and summarize earlier reports on its taxonomy, floral biology, processing of oil extraction, active constituents, uses, agronomy, breeding challenges and opportunities to the benefit of researchers, farmers and industrialists.

## 2. Taxonomy

**Figure 1:**
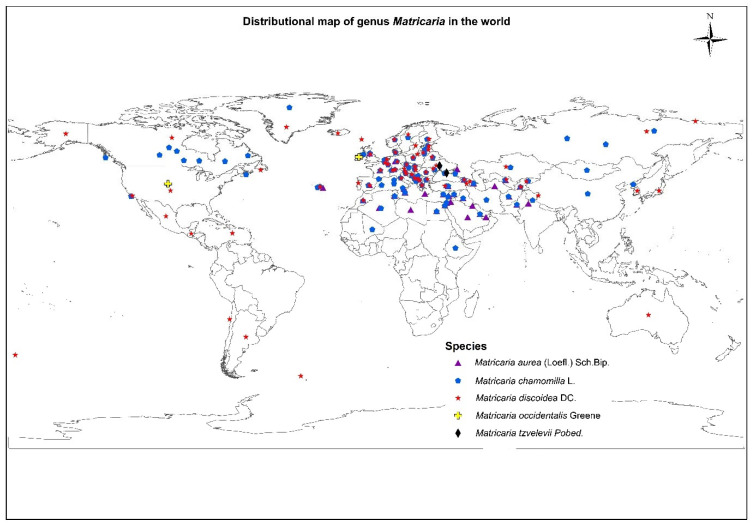
Accepted species of Genus *Matricaria* and their distribution.

*Matricaria* is a small genus (family Asteraceae), established by Linnaeus in 1753. Linnaeus chose the generic name perhaps due to its wide use in treating gynecological diseases, or “diseases of the womb (matrix)” [7]. The genus is characterized by 5 species, mostly distributed in Europe, northern Africa, Macaronesia, western, south-western and central Asia, and western North America [8]. With a wide range of geographical distribution and different adaption, the genus is usually found in disturbed land, grassland, areas along roads and railroads, in waste and vacant places [9,10]. Among the five recorded species, two species viz., *M. aurea* (Loefl.) Sch.Bip. and *M. chamomilla*, are found in India. The detailed species distribution of the genus *Matricaria* is provided in Figure 1 [11,12].

**Figure 2:**
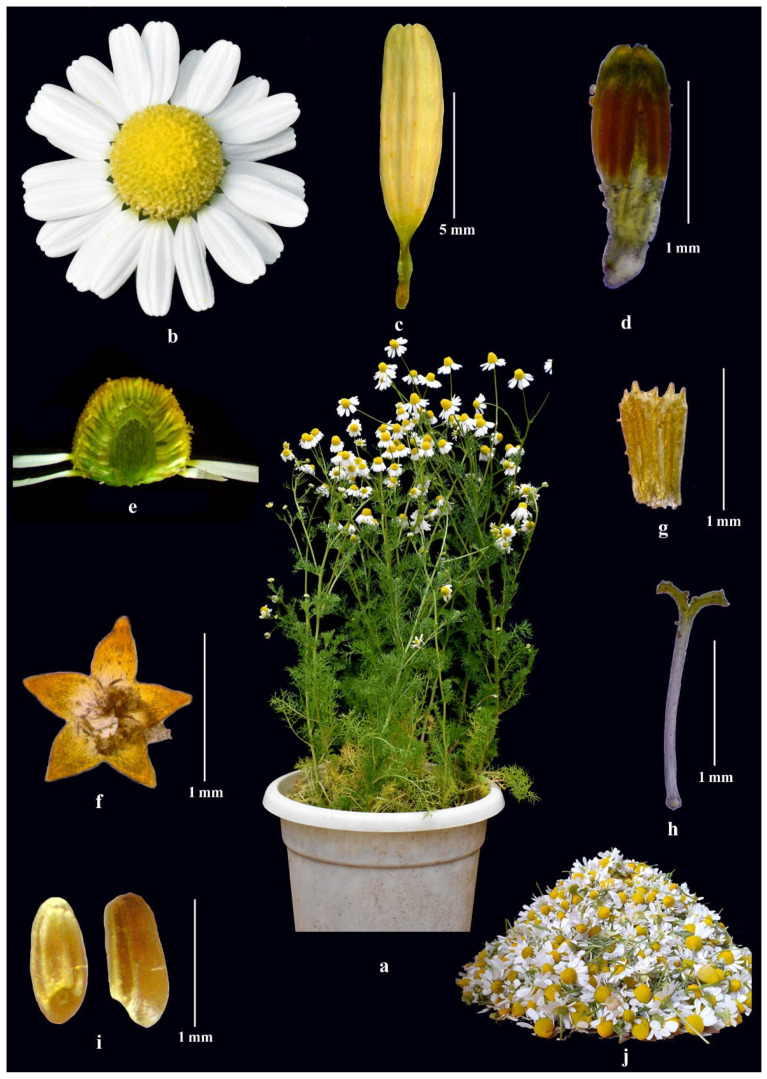
Different plant parts and habit of German chamomile: flowering plant (**a**); flower head (**b**); ray floret (**c**); disk floret (**d**); capitulum I (**e**); teeth/petals of disc floret (**f**); anthers (**g**); stigma (**h**); seed (**i**); fresh flower harvest (**j**).

German chamomile is often considered the original chamomile species. Its English name ‘chamomile’ originated from two Greek words ‘Chamos’ and ‘Melos’, which means ‘on the ground’ and ‘apple’, respectively. The latter probably refers to the unique apple-like fragrance of the flowers [6]. German chamomile is an annual, aromatic herb with a height of 10–60 cm. Sometimes, it can reach a height of up to 80 cm long. The plant has thin spindle-shaped tap roots and erect branched stems [13]. Its leaves are alternate and compound (Figure 2a). Flowers are arranged in heads or a capitulum, as the outer ring ray and inner disc florets, a common characteristic feature of family Asteraceae (Figure 2b,e). Heads are heterogamous, radiate, terminal and develop solitarily, with a diameter of 1–3 cm [4]. Peduncles are 3–6 cm long. The head is surrounded by cup-shaped involucres at their base [4]. The fruits it produces are called achenes, which are cylindrical, 0.8–1 mm long and around 0.5 mm wide, with 3 abaxial and 2 nearly marginal thin ribs (Figure 2i). An organized hierarchical scientific classification of German chamomile as per the Cronquist [14] is provided below:
KingdomPlantaeSubkingdom TracheobiontaSuper divisionSpermatophytaDivision MagnoliophytaClassMagnoliopsidaOrderAsteralesFamilyAsteraceaeGenusMatricariaSpecieschamomillaSynonyms*Matricaria recutita* L.;
*Chamomilla vulgaris* Gray
*Chamaemelum chamomilla* (L.) E.H.L.Krause
*Chrysanthemum chamomilla* (L.) Bernh

## 3. Chemistry, Processing and Ethanopharmacology

### 3.1. Bio-Chemical Active Constituents

Flower heads are the economically valuable element of German chamomile as they contain essential oil, composed of several important bio-chemical compounds. However, its leaf extract is also used in aroma-therapeutic proposes. The chemical composition of German chamomile varies from place to place, depending on soil, the specific environment and different genotypic backgrounds over the locations [15]. Some of the earlier studies on the secondary metabolites of German chamomile reported more than 120 compounds [16,17,18,19,20]. Among these active compounds, terpenoids (28 types) and flavonoids (36 types) are considered to be the important components classes. A systematic review on major chemical constituents, their composition in blue essential oil and population origin of German chamomile is presented in Table 1. 

### 3.2. Extraction of Essential Oil and Constituents

Fresh German chamomile flower heads produce a higher quality of essential oils than the dried flower heads, which may be due to drying, which causes a reduction in chamazulene and bisabolol contents [32]. Lower drying temperatures and shade-drying resulted in a maximum essential oil content while higher drying temperatures produced during oven, microwave, and sun drying decreased the essential oil content. The maximum and minimum chamazulene content were obtained using microwave and oven drying, respectively [33]. The essential oils of chamomile can be extracted from the flower heads at their near full bloom stage through hydrodistillation, steam distillation [34,35] and a subcritical CO_2_ fluid extraction process and microwave-assisted hydro-distillation [36]. Hydrodistillation is the simplest and cheapest distillation method, performed using a Clevenger-type distillation apparatus. Plucked flower heads are totally immersed in water at a 1:2 ratio (material-to-water) and boiled for 4 h in a round-bottom flask. Hydro-distillation of the fresh flower heads helps to identify essential oil compounds, such as (Z)-β-farnesene, D-limonene, and α-bisabolol oxide A [37]. The dried flowers of chamomile were subjected to hydrodistillation and analyzed with regard to their essential oil, reporting the presence of azulene-7-ethyl-1,4-dimethyl, bisabolol oxides A and B, limonene, *trans*-β-farnesene, bisabolone oxide, and isobornyl isobutyrate, which were identifies as essential oil compounds [17], while Amiri and Sharafzadeh, [22] reported α-bisabolol oxide A, chamazulene, en-yn-dicycloether, α-bisabolone oxide A, *n*-octanal, α-bisabolol oxide B, 1,8-cineole, α-terpineol and germacrene D as identified compounds. A study of essential oil from dried flower heads of chamomile, grown in Egypt, reported the presence of α-bisabolol oxide-B, chamazulene, bisabolol oxide A and bisabolol oxide as major compounds [38]. Dried flower heads of German chamomile were subjected to microwave-assisted hydrodistillation extraction, and 42.27% of α-bisabolol oxide A and 15.08% of chamazulene were identified, while in hydro-distillation both compounds were in lower concentrations, i.e., 7.97% and 1.67%, respectively [36].

There are several distillation processes such as continuous, quasi-continuous and batch steam distillation, which have been reported in the literature. However, batch steam distillation is the most popular process used in developing countries. Additionally, for obtaining the essential oil of German chamomile, naturally dried German chamomile heads can either be distilled or extracted in the solvent. However, longer heating periods required in the batch steam method resulted in high azulene and low bisabolol contents in the essential oils. Hence, the distillation time in water should be standardized to avoid longer water contact. To perform distillation, the material should be well crushed. The moisture content should be, on average, 80% (water) when using fresh German chamomile and 60% (water-alcohol) when using residues from German chamomile extraction. It is reported that chamazulene is not a genuine constituent of German chamomile, rather, it is formed as a result of dehydration, deacylation, and decarboxylation during steam distillation [39]. The dark blue color of high-quality chamomile essential oil is due to its chamazulene content [40,41,42,43,44]. Two different methods viz., the solvent extraction Clevenger distillation (SECD) and the standard Clevenger distillation method were employed for essential oil extraction of German chamomile flower heads. Solvents such as hexane, acetone, dichloromethane (DCM), ethyl acetate, and methanol were utilized for solvent extraction, and, among them, the highest essential oil yield was observed in DCM [45]. Another rapid method developed for the extraction of chamomile is subcritical CO_2_ fluid extraction and essential oil showed the presence of a matricine, α-bisabolol, dicycloether and farnesene compounds [46]. The subcritical water extraction (SWE) of essential oil in German chamomile was examined and it was observed that a temperature of 150 °C and a flow rate of 4 mL/min for 120 min resulted in more valuable essential oil with regard to the oxygenated compounds [47]. 

The active ingredient profile, which displays the characteristic of German chamomile oil, is not obtained by distillation because distillation largely decomposes the therapeutically important thermolabile spiroethers. However, the advantage of distillation is that it allows for an almost complete extraction of the other components of the essential oil. Additionally, it is highly challenging to obtain the essential oil of German chamomile by extraction. Thus, in extracting essential oil, using 45% ethanol, only about half of the bisabolol and chamazulene-containing oils in the product are recovered in the extract with the sensitive spiroethers. Steam distillation leads to a high content of thermolabile spiroethers in the essential oil. The preferred parts of the German chamomile plant, for aqueous distillation or steam distillation, are German chamomile flower heads and stalks [48]. For extraction, German chamomile flower heads are the preferred parts of the German chamomile plant [49]. As mentioned above, the extraction of fresh, dried, or deep-frozen German chamomile flower heads is preferably carried out using an aqueous organic solvent, such as ethanol, isopropanol, and methanol, etc. The water content should, preferably, be no less than 20% (*v*/*v*). In this way, German chamomile oil with a high content of natural Spiroethers can be obtained. The method of extraction should be economically viable, and should best utilize the residues of German chamomile. In addition to the improved yield of the active ingredient, this should lead to considerable savings in the cost of raw materials for manufacturing extracts, thereby reducing the overall cost of operation. The distillate obtained should be collected in a vessel that allows phase separation, based on the different specific gravities between the essential German chamomile oil and water. The essential oil obtained can be used without further purification, for the manufacture of German chamomile extract preparations. Distillation may be performed in a conventional mobile or stationary distillation vessel.

### 3.3. Ethanopharmacological Properties and Uses

The above given active constituents of blue essential oil of German chamomile bear more than 20 pharmacological properties, making this plant a star herb (Table 2). Among them, anti-allergic, anti-spasmodic, anxiolytic, anti-inflammatory, anti-microbial, neuroprotective, antioxidant, anti-cancer and hepatoprotective are the major pharmacological activities reported. Chamomile extracts are capable of regulating tumor angiogenesis by down regulating in a dose-dependent manner [50]. Chamomile tea is also used in traditional home remedies to treat several stomach disorders, body aches and to reduce stress. The essential oil is also used as a therapeutic and aromatic component in bath soaps, shampoo and other hair-care formulations. Additionally, chamomile extract, essential oil and dry flower heads are use in the food industry to add flavor, colour and taste to food products. Interestingly, a recent study on poultry feed showed that the dietary addition of 1 g chamomile flower powder per kg of feed during the laying period improves productive performance, net revenue and the relative economical returns, and reduces feed consumption [51]. This study has attracted the interest of researchers in further studies on chamomile’s uses and its commercialization at the dairy and poultry level. A systematic review on its pharmacological activities and their utilization is provided in Table 2.

## 4. Genetic Improvement

### 4.1. Breeding Challenges

Presently, several diploid and tetraploid varieties of German chamomile are available worldwide, but most of them are old local collections and mixed heterogeneous populations. These cultivars lack favorable agronomic traits for machine harvesting. The uniform pick length of flower heads, synchronous flowering time and uniform flowering flushes present some major challenges in the large scale cultivation of this plant, all of which could be improved to make flower harvesting simpler, viable at a low cost and to facilitate mechanical harvesting. Additionally, the dry flower yield under machine harvest is still unexplored. Furthermore, their small flower size, high seed shattering and uneven anthesis flushes cause problems in traditional plant-breeding techniques for the genetic improvement of German chamomile. The hybridization tools such as self-incompatibility and male sterility are not yet reported in this crop, however, some degree of self-incompatibility (25–28%) in diploid cultivars and pollen sterility (10.20%) after intra-specific crossings were reported in German chamomile [87].

### 4.2. Reproductive Biology

German chamomile flower head has two types of florets as pistilate ray florets (Figure 2c) and bisexual disc florets (Figure 2d). The ray florets are narrowly winged with white corolla. The deflexed laminas of ray florets are 6–15 mm long and 2.5–3.5 mm wide [7]. The number of ray florets varies between 16–24, while disk florets are numerous and the corolla is tubular, campanulate, yellow or greenish yellow, 1.5–2.5 mm long and 0.5 mm broad having five teeth present at end of tube (Figure 2f) [12]. Anthers are linearly bi-lobed and dehiscing longitudinally at the time of anthesis (Figure 2g). The anthesis in German chamomile last long up to 10–15 days. The anthers dehiscence occurs as first flush of anthesis which is overlapped by stigma receptivity flushes in ray and disc florets. The movement of stigma receptivity flush is generally taking place in upward direction towards tip of flower head; but sometimes it was observed to be start near center and move towards both the directions. Minute size of floret (2–3 mm) make hand emasculation impossible in German chamomile. However, the polygamous condition may help in controlled pollination by practicing disc removal of the female bud in a crossing pattern.

### 4.3. Genetic Diversity 

The studies on genetic diversity assessment in German chamomile are very scanty. Only a few reports are presented in literature on morphological and molecular marker based genetic variation in this crop. Solouki et al. [88] conducted a Duncan test for 24 different traits in German chamomile in terms of economical yield, number of flower heads per plant, and essential oil content, which were found to have maximum coefficient of variance, respectively, whereas the diameter and height of flower heads showed a minimum diversity variance coefficient. To analyze the genetic diversity of German chamomile among 25 populations from Iran, 29 RAPD primers were used, and 369 bands were detected of which 314 (85.09%) were found to present high polymorphism [88], Furthermore, during a study of 25 German population using 18 RAPD markers, 93.18% polymorphism (205 fragments out of 220) were recorded [89]. Additionally, Wagner et al. [90] analyzed the genetic diversity in di- and tetraploid German chamomile using 18 RAPDs and 3 AFLP molecular markers and characterized all the germplasm into two major clusters by their origin of germplasms. Otto et al. [91] reported less genetic diversity among tetraploids than in diploids using (Single Nucleotide Polymorphisms) SNPs based on genotyping by sequencing (GBS). An ISSR analysis was found to be the most economical among PCR based markers, and using this technique 85.4% polymorphism were recorded in 15 genotypes of chamomile using 5 ISSR primers [92]. Nei’s genetic diversity index (h) has also been calculated to obtain average gene diversity per locus in German chamomile of Iran. Different values of Nei’s genetic diversity index are reported by different researchers, ranging from 0.16 to 0.36 and 0.23 to 0.528 [89]. Tsivelika et al. [93] reported a great variability between eleven native populations of Greek, and five commercial varieties. The study showed that the local native populations were more stable for phenotypic traits and had a high variation for a-bisabolol and chamazulene; hence, these populations could be used for future breeding purposes. Ahmadi et al. [94] assessed the genetic variation of 23 chamomile populations with 10 ISSR primers which resulted in four major groups being classified with respect to the geographical distribution of the germplasm. The study showed that European and Iranian populations are the most diverse populations in terms of phenotypic traits of interests. Another study by Adeli et al. [95] concluded that the Esfahan population produced a higher essential oil percentage and oil yield. Italian populations differed significantly for plant height and flower yield [96]. Singh et al. [97] reported a wide range of genetic diversity through a D^2^ analysis of dry flower yield, flower diameter, disc floret periphery, disc floret height and the number of flower heads per plant in 40 Indian chamomile populations. Lal et al. [98] generated a novel variation in polygenic traits of chamomile through gamma radiation and identified a mutant with higher essential oils for commercial cultivation. 

Molecular markers proved to be the most robust method for plant breeders in the classification of genetic diversity, organization of germplasm, selections at genomic level, selection at early stage and selections for destructive traits, etc. More specifically, German chamomile molecular markers could help in the assessment of genetic diversity and early selections for its flowering traits. The most extensively explored molecular markers so far in German chamomile are RAPD, AFLP and ISSR [88,89,90,92,94]. However, a few studies also reported on the use of SNPs for gene mapping and population structure in German chamomile [91,99]. Ahmadi et al. [94], showed that an ISSR molecular marker-based classification was not in accordance with the morphological trait-based categorization of the germplasm, which showed a poor correlation of the studied primers with phenotypic traits. Wagner et al. [90] identified three AFLP-markers associated with (−)-α-bisabolol locus through Bulk Segregating Analysis (BSA). They also reported 17 AFLP and one RAPD markers linked with chamazulene content in the blue essential oil. These molecular markers could be used in pre-flowering selections in German chamomile. However, studies on Genome-wide association mappings using new molecular markers such as SNPs are urgently needed to facilitate the genetic improvement of German chamomile through genomic and marker-assisted breeding. 

### 4.4. Breeding Approaches 

German chamomile is a allogamous crop and small flies are the major pollinating agents. Higher dry flower yield, synchronous flowering, a high oil content and better quality blue essential oil are essential. A systemic implication of traditional breeding techniques requires an understanding of the breeding behavior of the crop. Earlier reports showed 82–100% seed formation under selfing and open pollination conditions [87]. However, reports on the development of advance generations and trait inheritance are scarce. The inheritance of most of the traits in this plant have not yet been studied. Few traits controlling chemical components and contributing towards essential oil quality were studied and revealed a single recessive gene for (−)-α-bisabolol and chamazulene [90]. The flower yield and oil content may consider quantitative traits and could be improved through a direct and indirect selection of component traits such as number of flower heads, days to flowering and flower size, etc. A study in this direction by Golparvar and Pirbalouti [100] showed that the indirect selection for number of flower heads per plant and days to 50% flowering could improve dry flower yield and essential oil percent in German chamomile. They demonstrated that oil content has a positive correlation with dry flower yield and crop canopy. Pirkhezri et al. [89] also reported positive associations of oil content with the number of flower heads and flower yield, which showed that both oil content and flower yield could be improved simultaneously in a breeding program, while oil content was negatively correlated with days to maturity, which indicated that the early and optimal flower harvesting stage is important in chamomile [89,95]. Particularly, a selection from local landraces and polyploidy breeding are the most often used methods of varietal development and improvement for flower yield and oil in this crop. Researchers developed tetraploids through colchicine treatment to seeds in German chamomile [101]. The flower diameter and weight were higher in tetraploid cultivars [96]. However, plant height and flower-pick length increases in tetraploids, which could cause difficulties in machine harvesting. Attempts has been also made to develop triploid chamomile for commercial cultivation using diploid and tetraploid parents in crossing [102]. However, seeds of triploid chamomile hybrid depend on the availability of any hybridizing tools such as male sterility and self-incompatibility, etc. Diploids and tetraploids were found to be statistically similar on oil percentage [96]. Albrecht et al. [103] composited 30 lines from progeny test-crossing, based on single plant selection for up to four generations (L1−L4 generations) and developed an improved variety of German chamomile. 

A gene transfer technology has been developed in German chamomile by using cationic carbon nanotubes (CNTs), with a high interaction ability with DNA that efficiently protects it from damage occurring as a result of digestive and ultrasound enzymes. The fluorescence microscope also revealed the potential of nanoparticles in transferring the ssDNA-FITC, and the simultaneous utilization of CNTs and ultrasound significantly improved the transfer efficiency of ssDNA-FITC to German chamomile cells [104]. Terpenoid biosynthesis pathways analyses were carried out in German chamomile (*M. Chamomilla*) and Roman chamomile (*Chamaemelum nobile*) by gene co-expression networks and revealed that higher terpene synthase expressions correlated with sesquiterpenoids in German chamomile, identifying amore sesquiterpenoid concentration. Volatile compounds contain unigenes which were significantly enriched in plant-pathogen interaction pathways, thus influencing the volatile compounds of German chamomile and providing higher resistance to pathogens because it contains ten times more unigenes related to plant-pathogen interactions than Roman chamomile [105]. A cloning and functional analysis of three aphid alarm pheromone genes was established in German chamomile (*M. chamomilla* L.) which studied the in vitro and in vivo enzyme activity of three sesquiterpene synthases. A hairy roots transformation system was also developed, and it was found that the over expression of sesquiterpene synthase genes resulted in the accumulation of γ-muurolene in hairy roots. In biosynthesis and the regulation of volatile terpenes, the action of aphid alarm pheromones forms the basis for molecular study. qPCR signified high expression of these genes in young flowers and showed higher correlation with amounts of essential oil compounds viz., (E)-β-farnesene, β-elemene and germacrene D [106]. Mutation breeding with chemical radiation mutagens, mass selection and polyploidization breeding were used as the major conventional breeding techniques for German chamomile improvement. Nowadays, biotechnological approaches to create fingerprints of genotypes and to explore relationships of species have also been established [99]. Additionally, germplasm conservation at seed banks as well as field gene banks may be considered as a possible method by which to avoid over-collecting from the wild and the loss of genetic resources. Still, uniform flowering, equal pick length, higher essential oil genotypes and additional yield using suitable agrotechnologies are some of the crucial areas that require attention in future German chamomile studies. Several researchers aim to genetic engineer the growth-process of the plant to improve secondary metabolites [107,108].

## 5. Agro Technology

An increase in the demand of chamomile oil on the international market during the last two decades has encouraged farmers to replace the cultivation of traditional crops with German chamomile. However, this annual plant requires special attention especially during the harvesting of flowers, as the flower heads do not mature for harvesting at a single picking, and require more labor for further pickings. Similarly, for higher flower and oil yield, the crop requires suitable agronomic practices including high yielding cultivars, optimum crop geometry, proper nutrient and water management, insect pest control and proper harvesting methods.

### 5.1. Climatic Conditions

German chamomile can be grown successfully across a wide range of soils and climate [109]. However, long, warm days with cool nights and well fertile soil with good topsoil are preferred by the plants. However, the essential oils and azulene content are greatly influenced by temperature and sunshine hours as opposed to soil type [110]. Greater day lengths are reported to increase oil content and quality as well as chamazulene content. The plant can withstand low temperatures of −10 °C during night time, however, thrive in full sun with 15–20 °C day temperature during the vegetative phase. For good seed germination, the temperature should range between 10 °C and 20 °C. Furthermore, being an undemanding crop that is tolerant to soil alkalinity, it can be grown on poor loamy soil and can be suitable for cultivation in saline soils and prove to be sustaining in water deficit conditions [111]. The crop is grown during late September or early October in plain regions, and in the second fortnight of December in the North Indian hills, respectively [112]. Similarly, in eastern India, the first week of December is the best sowing time to obtain higher yield [113]. Climatic stress affected the essential oil yield in German chamomile. Cold stress conditions were examined to observe the response of German chamomile genotypes at flowering stage and indicated that the essential oil content increased with a decrease in temperature from 25  °C to 10  °C, however, a further increase in cold stress decreased the essential oil [114]. The foliar application of proline at the rate of 100 mg per liter successfully enhances growth under water stress and is recommended to improve the yield of German chamomile in water deficit conditions [115]. Additionally, cow fertilizer in the severe stress and biosulfore in mild stress conditions were found to be effective in improving chamomile production [116]. Bio-polymer chitosan is an important natural material that is used to overcome the damage of water stress in chamomile and enhance chamazulene content [117]. The foliar application of chitosan at the rate of 40 ppm at flowering stage enhanced chamomile flower number, weight per plant and yield [118]. Plant growth regulators may be used to achieve physiological uniformity for machine harvesting in German chamomile. However, the effect of different plant growth regulators should be tested in future studies. The application of salicylic acid at the rate of 50 ppm improves dry flower and essential oil yield in German chamomile [119]. Flower as well as essential oil yield are higher in early sowing, while a delay in sowing improves α-bisabolol oxide A [119]. This negative correlation of content and composition of the essential oil with date of sowing should be further dissected in future studies by developing an understanding polygenic traits involved.

### 5.2. Propagation

German chamomile can be grown directly through seeds or by raising seedlings in a nursery or vegetatively by use of cuttings [120]. However, direct sowing and transplanting-seedling methods are generally preferred by farmers. Both methods have their own advantages and disadvantages; since direct sowing is much easier and cost effective but also much riskier, as maintaining optimum temperature and humidity in field conditions is very difficult, which results in poor germination and poor crop stand. On the other hand, raising a seedling in a nursery bed requires more labour and hence is a costlier method. However, optimum conditions are provided in the nursery bed which results in healthier plants in a smaller area. Furthermore, these seedlings have a very negligible mortality rate during transplanting and results in good early growth.

#### 5.2.1. Through Seeds

The seeds of German chamomile are very small in size; having a thousand seed weight of 0.088–0.153 gm [4]. So, the seed should be mixed with sand (1:10 ratio) for easy sowing, furthermore, seeds stay well in the soil and losses due to insect and wind, etc. are minimized. Around 2.0–2.5 kg seed per hectare is enough when sown at a row spacing of 30–40 cm [121]. The seed should be sown in line at a depth of no more than 2 cm in the soil. Mounir and Gilles [122] suggested that the seed requires light for germination and hence should be cover lightly with soil. In contrast, Timothy and Mwangi [120] reported that light did not have any effect on seed germination and sowing can be performed directly without soil covering. To get achieve better germination, a light irrigation after sowing should be applied to obtain optimum moisture conditions, without which a poor and patchy crop stand is obtained. Generally, seeds germinate within 2–3 weeks and when seedlings attains 5 cm height thinning should be performed to obtain the desired population. To maintain optimum moisture in the field, light irrigation should be provided regularly.

#### 5.2.2. Through Seedlings

The direct sowing method generally results poor or patchy germination, hence the transplanting of seedlings is preferred [4]. For raising seedlings in a nursery of one-hectare land, around 0.50–0.75 kg clean seeds, with a good germination percentage, is sufficient in an area of 250–300 m^2^ [2,4]. The soil must be loose and friable, loam to sandy loam in texture, humus rich and well drained for optimum growth and the development of seedlings. Raised nursery beds of a suitable length and 1 m width in size should be prepared by breaking the clods, removing stones, weeds and mixing a sufficient amount of well rotten poultry or farm yard manure in the soil. The seeds of chamomile should be sown at a depth of 1–2 cm in soil with a row spacing of 8.0 cm and covered with fine soil followed by light irrigation [2]. To maintain optimum moisture in the nursery beds, light irrigation should be provided as and when needed. After sowing the nursery, seed germination starts in 4–5 days and seedlings become ready for transplanting in the field after 4–5 weeks [123].

#### 5.2.3. In Vitro Propagation

Since the propagation of German chamomile from seeds is relatively easy, the tissue culture techniques applicability mainly focused on certain aims, such as efficient secondary metabolites production, superior individual clonal propagation in uniform genotypes, for cryopreservation and breeding purposes. A variety of research has been reported for the production of valuable secondary metabolites [124,125], cryopreservation [126], and clonal mass propagation [127]. A shoot primordial mass is considered as most appropriate for the vegetative propagation of plants because of its high regenerative potential, growth rate, and genetic stability [128]. An in vitro method was developed for the micropropagation of chamomile with an appropriate amount of an MS (Murashige and Skoog), liquid medium supplemented with 2 mg/16-benzylaminopurine (BAP) and 3% (*w*/*v*) sucrose [129]. 

The effect of growth regulators and explants types were studied on embryogenesis and rooting of German chamomile and growth regulator viz., NAA (naphthalene acetic acid) and kinetin at different levels, which demonstrated the production of embryogenic calli from leaf explants, stem explants, and the axillary bud explants in a culture medium containing (NAA: 0.5 + Kin: 1 mg/L) hormonal composition, while a culture medium (NAA: 1 + Kin: 0 mg/L) developed the highest rooting [130]. Another in vitro study was conducted to study the effect of additives (NAA and BAP, i.e., 6-Benzylaminopurine) on callus tissue formation of chamomile explants and cultivar to determine the optimal growth-regulator content for callus tissue initiation. The initiation of callus, using leaf explants with the combination of auxin NAA and BAP is possible in chamomile using MS proliferating medium enriched with NAA and BAP¸ developing a large callus tissue mass at a rate of 3 mg dm^−3^ and 7 mg dm^−3^, respectively [131]. A protocol has been developed for the induction of somatic embryogenesis from whole inflorescence explants of *Chamomilla recutita* L. (chamomile). In vitro propagation in chamomile is possible and can be used to increase the overall yield and chamazulene content present in the capitula of flowers [132]. Different concentrations of auxin viz., NAA and kinetin, were utilized for callus induction and growth in Chamomile’s explants, cultured on a basal MS medium containing growth regulators. Axillary bud and stem explants have presented the highest percentage of callus induction obtained in Kinetin (1 mg L^−1^) and NAA (1 mg L^−1^) treatment combination. The different combinations of NAA and Kinetin significantly affected the callus volume derived from different explants and produced maximum average callus volume (17.49) at Kinetin (1 mg L^−1^) and NAA (1 mg L^−1^) treatment combinations [133]. Recently, Hosseini et al. [134] showed that benzyl amino pourin (4.4 µM) and indole acetic acid (2.2 µM) in MS media provided the highest regeneration percentage (93%) for both hypocotyl and cotyledon explants.

### 5.3. Field Preparations 

For the cultivation of German chamomile, the field should be ploughed two to three times with mouldboard plough to obtain a levelled field with optimum tilth. Furthermore, all the weeds and stubbles of previous crop should be removed from the field. If sufficient moisture is not available in the soil, pre-sowing irrigation should be applied. Rotten poultry manure or farm yard manure or vermi-compost should be thoroughly mixed in the soil before last ploughing to make the soil friable. In the case of transplanting the seedlings, farm yard manure should be applied 30 days before the transplantation in the field [2]. During the vegetative phase, light hoeing is enough to make soil loose and weed free for the better growth of the crop.

### 5.4. Plant Geometry 

The arrangement of plants in different row spacing, spatial arrangements and plant density plays a vital role in utilizing the natural resources and affects the final flower yield and oil content of German chamomile. Row spacing of 10 to 80 cm has been reported to be optimum in different condition [135,136]. Flower heads yield per hectare increases with a decrease in row spacing, while the yield per plant increases with increase in row spacing. However, essential oil content and azulene were not affected by row spacing. Tadesse and Chala [137] recorded maximum dry flower heads yield of 517.2 and 586.7 kg/ha in Ethiopia during the first and second years, respectively with 40 × 40 cm crop geometry. Similarly, Kanjilal and Singh [113], in Assam (India), recorded higher yields of German chamomile when sown at a spacing of 30 × 30 cm in the first week of December. The highest crop yield was obtained with a wider row spacing of 35 and 45 cm in Lublin, Poland when sown in the month of April [138]. In Iran, Pirzad et al. [139] obtained the highest dried flower heads yield (1241 kg/ha), seed (765 kg/ha), essential oil (8057 g/ha) and total biomass (2716 kg/ha) at a spacing of 10 × 30 cm while the harvest index of essential oil was found highest with 5 × 30 cm spacing. However, Arslan et al. [140] from Turkey reported that narrow row spacing of 15 cm yielded the highest gross profit (EUR 8818.33 ha^−1^) using Zloty Lan cultivar and further increased row spacing, decreases profits considerably. Similarly, the highest yield of flower heads was obtained at a narrow spacing of 15, 20 and 30 cm [141,142,143]. Dutta and Singh [143] also recorded highest fresh flower heads yield and oil content under 30 cm^2^ spacing.

### 5.5. Intercropping 

One of the main objective of intercropping is to produce more crops per unit area without competing for resources viz. space, water, nutrients or sunlight. German chamomile can be grown as an intercrop with celery, ajwain, fennel and sowa at a 1:1 ratio which is found to be suitable for palmarosa and lemongrass (dormant in winter) [144]. Jahan and Jahan [145] conducted an experiment in Iran and observed a reduction in proportion of seeding rates of German chamomile with Pot Marigold from 100:0, 75:25, 50:50, 25:75 and 0:100 occurs, and that the leaf area, total dry biomass and seed yield of Chamomile decreases proportionally while, chamazulene content increased significantly. However, Naderidarbaghshahi et al. [146] reported no differences in chamomile yield when intercropped with saffron (inter-row spacing of saffron 30 cm and chamomile 10 cm) or when grown as sole crop. Similarly, the herb and flower heads yield of chamomile was not affected by growing it as a companion crop with lemongrass [147].

### 5.6. Nutrient Management

Generally, German chamomile is a low demanding crop, but for commercial cultivation it requires a balanced nutrient supply throughout its crop cycle which results in higher flower heads yield and oil content. Good quality farmyard manure, poultry manure or vermicompost at 10 t/ha, depending upon the soil fertility, should be applied before transplanting/sowing of the crop. It has been reported that chamomile responds well to the application of nitrogen (N_2_) as compared to phosphorus (P) and potassium (K) [4]. Being a major plant nutrient, N_2_ encourages the plant vegetative growth of chamomile and increases its essential oil yield [148,149]. Rahmati et al. [150] have also reported a positive effect of N_2_ on plant root characteristics, chamazulene and essential oil content. Increasing the application of N_2_ from 0 to 60 kg/ha in two split doses, i.e., as a basal dose and one month after transplanting the seedlings resulted in higher growth parameters and yield of chamomile [151]. However, Hamzeii et al. [152] from Iran reported the application of N_2_ at 150 kg/ha as an optimum dose for an increased number of flower heads and dry flower heads yield per plant, harvest index and essential oil content as compared to 75 and 225 kg/ha. Giannoulis et al. [153], from Greece, also recorded higher dry flower heads yield and essential oil yield (3800 and 25 kg/ha, respectively) with the application of 160 kg/ha N_2_ under irrigated conditions. On the other hand, Upadhyay et al. [2] obtained the highest dry flower heads yield (4.11–4.14 Mg/ha), oil yield (37.32–37.84 k/ha) and oil content yield (0.91%) with the combined application of N, P_2_O_5_ and K_2_O at 100:60:40 kg/ha and when the fertilizer was incorporated in the surrounding soil of plants. Out of which, 1/3rd N_2_ and the entire quantity of P_2_O_5_ and K_2_O was applied as basal dose and 1/3^rd^ of N_2_ at 25–30 days after transplanting (DAT) and 40–45 DAT. Similarly, the highest yield was obtained with the application of a higher doses of N_2_, P_2_O_5_ and K_2_O at 120:50:50 kg/ha by Balakram et al. [154]. Mukesh et al. [155] reported that the interaction of N_2_ (210 kg/ha) + sulphur (60 kg/ha) + spacing (40 cm) resulted in increased plant height, plant spread, number of branches and flower heads per plant and total number of flower heads per plot. 

However, continuous application of chemical fertilizers affects the soil microbial biomass, which in-turn affects the soil productivity. On the other hand, organic manures viz. farm yard manure (FYM), vermicompost and poultry manure not only improves soil physiochemical properties but also improves the yield quality [156]. Kumar et al. [148] reported that the application of farm yard manure at 20 t/ha resulted in the highest fresh (41.91 kg/ha) and dry biomass (9.25 kg/ha) of the plant at the flower bud initiation stage. Similarly, the use of poultry litter at 12.5 kg/m^2^ resulted in the highest capitula fresh mass (2483.7 kg/ha) and dried mass (471.8 kg/ha) as compared to 0, 2.5, 5.0, 7.5 and 10.0 kg/m^2^ [157]. Jimayu [158], during his study on the application of vermicompost on chemomile, reported that after an increase in the amount of vermicompost from 0 to 20 t/ha, the flower heads yield increases nonlinearly and resulted in the highest dry and fresh flower heads yield (653.8 and 3335.7 kg/ha, respectively) with an application of 20 t/ha. The positive effect of vermicompost on early flowering, plant height, flower heads yield, chamazulene and essential oil content have been reported by Hadi et al. [159]; Azizi et al. [33]. Furthermore, the application of all compost + liquid compost increases the diameter of the flower head, fresh or dry weight of flower heads and the essential oil content of chamomile over chemical fertilizers [160]. In contrast, Jahan and Jahan [145] recorded no significant differences in the chamazulene content with the application of animal manure from 0 to 50 t/ha. 

The integration of chemical fertilizer with organic manure has been reported to enhance the crop yield and also improves the qualitative and quantitative characteristics of chamomile. The substitution of chemical fertilizer by vermicompost, i.e., 75% N_2_ through ammonium nitrate (135 kg/ha) and 25% through vermicompost (3 t/ha) have been reported to enhance the fresh and dry flower heads yield (7539.45 and 1715.93 kg/ha, respectively) and essential oil yield (6.95 kg/ha) [161]. Similarly, Prasad et al. [162] recorded higher dry flower heads yield with the application of 50% of the recommended dose of fertilizer (RDF-60:40:30 kg/ha) + 5.0 kg/ha PSB + 5.0 kg/ha azotobacter which further resulted in a maximum net return (Rupees 1,31,341) and benefits in terms of cost ratio (2.09). Irrigation and nitrogen fertilization affected essential oil production and the composition of German chamomile; lower essential oil and increased α-bisabolol and chamazulene content with increased irrigation and nitrogen fertilization, respectively [153]. 

### 5.7. Water Management 

Chamomile has a shallow root system and therefore, does not exert moisture from the deeper layers of the soil. To obtain a good yield, frequent irrigation is required to maintain optimum moisture level, and to avoid flooding. Giannoulis et al. [153] from Greece reported a significant effect of irrigation on the crop yield of chamomile and obtained higher fresh and dry flower heads yield (5250 and 2200 kg/ha, respectively) as compared to in a rainfed condition but, the essential oil yield was lower. Irrigation during the flowering stage increased the flower heads yield in addition to one extra flush of flower heads with a delayed seed formation [4]. Kerches [110] also recorded an increased yield when irrigation was applied at the rosette stage. During the whole crop life cycle, about 4–6 irrigations should be applied. However, more frequent irrigations (about 6–8) are required to raise the crop on alkaline soils [163]. 

### 5.8. Weed Management

Weeds present the major problem in chamomile crops and reduces dry flower heads yield by as much as 34.4% as compared to weed-free conditions. In general, 3–4 weedings with manual labor are sufficient to raise a healthy crop in normal soil, however, this method is expensive for growers. In the chemical control of weeds, Kewalanand and Pandey [164] recommended oxyfluorfen at 0.4 kg/ha and pendimethalin at 1.5 kg/ha to be most economical and as a safe way to manage weeds in chamomile in Pantnagar conditions. Moreover, oxyfluorfen at 0.4 kg/ha and weed-free conditions resulted in significantly higher dry flower heads yield and oil yield of chamomile. Similarly, the application of sodium salt of 2,4-D (2,4-dichlorophenoxyacetic acid) at 1.0–1.5 kg/ha after four weeks of transplanting resulted in a good control of weeds [4]. However, crops treated with herbicides results in lower chamazulene content, and led to a lower bisabolol content in the second harvest of the crop due to the interference of herbicides with the metabolism of the secondary products. In the organic cultivation of chamomile, Kwiatkowski et al. [121] from Poland reported around a 20% reduction in the number of annual weeds following the application of bioproducts Bio-algeen and Herbagreen Basic, as compared to control treatment (without application of bioproducts). They also observed a decrease in the total number of weeds alongside a greater diversity of weed species with foliar application of these bioproducts, while a greater number of weeds with lower biodiversity was observed in control treatment. In another study, Frabboni et al. [35] applied essential oil of rosemary (*Rosmarimum officinalis* L.) and oregano (*Origanum vulgare* L.) at two concentrations, i.e., undiluted and 50% diluted, three times during the chamomile crop cycle and observed higher weed control efficiency under undiluted essential oil treatment. In a different study, Kwiatkowski et al. [121] reported that crop geometry significantly influences the weed infestation, and found a reduced weight of weeds under narrow row spacing (30 cm) as compared to wider spacing (40 cm). To manage weed infestation in salt-affected soils, hand weeding/hoeing performed once, one month after transplanting is sufficient, as the plant smoothers the weed once establish [165]. Furthermore, it has been reported that weed management during 5–11 weeks after transplanting assists in obtaining higher flower heads and oil yield of chamomile [166].

### 5.9. Plant Protection

German chamomile has been reported to be affected by as much as 26 insect pests in Perugia, Italy conditions and majority of them cause damage by feeding on vegetative or reproductive organs, e.g., Miridae, Thripidae, Pseudococcidae and Pentatomidae families [167], while Noctuidae, Elateridae and beetles causes tunnels on heads, roots and leaves, respectively. The researchers also reported *Anaphes fuscipennis* and *Telenomus eumicrosomoides* parasitoids in chamomile. In Indian conditions, Mathur and Sharma [168] observed shedding of flower heads by *Nysius minor* and defoliation of plant by *Autographa chryson* insects. For control of black aphid (*Doralis fabae* Scop.) and *Ephestia elutella* Hb, application of fosfothion (0.2%) and fumigation with methyl bromide @ 3 kg/100 m^3^ have been recommended [4]. Similarly, major diseases and fungi reported are white rust (Pathogen: *Albugo tragopogonis*), powdery mildew (Pathogen: *Erysiphe cichoracearum*), leaf blight (Pathogen: *Alternaria* spp.), *Halicobasidium purpureum*, *Puccinia anthemedis*, *Phytophthora cactorum*, *Peronospora leptosperma*, *Cylindrosporium matricariae, Septoria chamomile* which causes damage to chamomile. For management of leaf blight incidence in early March, application of Benlate (0.1%) is effective.

### 5.10. Harvesting and Yield

**Figure 3:**
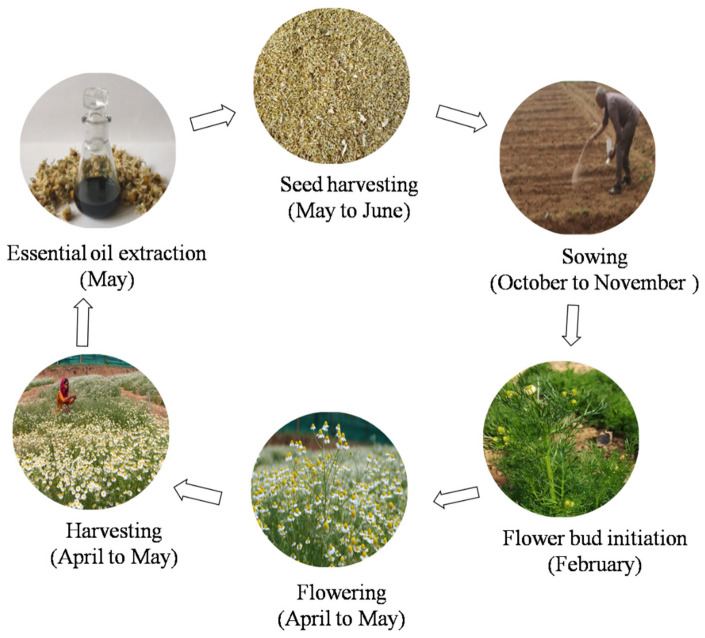
Crop growth cycle of German chamomile in mid hills of western Himalayan region.

Depending upon the soil and climatic conditions flowering starts in the month of March and continues up to April. Complete crop growth cycle in mid hills of western Himalaya has been illustrated in Figure 3. Flower heads are produced in 4–5 flushes; the 2nd, 3rd, and 4th flushes are the major contributors to flower heads yield [4]. However, harvesting/picking of flower heads is most laborious operation in its cultivation and accounts major portion of the cost of cultivation. The peak period of plucking is between 2nd week of April and 3rd week of May in lower Himalayan range of Northern India (Personal communication). Seeds of German chamomile are generally sown in September and harvested in March end to mid-May in northern India (Personal communication) and in February to April in sandy soils of Egypt [38]. Care should be taken for harvesting of the flower heads as quality varies with different stages of flower (buds, semi-opened buds, flowers) and near full bloom stage have been reported to result in best quality of produce [4]. After harvesting of flower heads, drying under shade for 3–4 days are recommended before distillation. Mishra et al. [144] recorded highest oil content (0.44%) and farnesene content (15.16%) in oil when flower heads were shade dried, while sun dried flower heads resulted in highest chamazulene (22.77%) besides poor oil yield. Under normal soil conditions, crop can yield 6.0 t/ha fresh and 1.0–1.5 t/ha dry flower heads [112] while, in saline–alkaline soils, the crop can yield 3.75 t/ha fresh flower heads [142]. Further, the potential yield can be enhanced by developing commercial hybrids by exploring male sterility in German chamomile [169].

## 6. Trade and Adulteration 

*M. chamomilla* is allied to *Anthemis cotula* L., which is a poisonous plant with a revolute smell and thus leads to confusion in identification. In the Unani system of medicine, three species viz. *Matricatia recutita* L., *Anthemis nobilis* L. and *Corchorus depressus* L., are reported under same vernacular name, i.e., ‘Babuna’, which created confusion during the identification of the species. Ghauri et al. [170] attempt to solve the ambiguity among the species by studying taxonomic and anatomical elements, and found that the name ‘Babuna’ belongs to *M. chamomilla* whereas *A. nobilis* L. and *C. depressus* are used as an adulterant. Various methods were developed with near infrared spectroscopy in combination with chemometrics to detect, quantify and authenticate some of the common toxic adulterants of German chamomile. Mahgoub et al. [171] successfully constructed several near-infrared spectroscopy models for the detection of chamomile and its toxic adulterants. A soft independent modelling of class analogy and the orthogonal projection of latent structures models were used for sample authentication with 100% sensitivity, while Partial least squares models showed high predictive power for each individual adulterant in adulteration mixtures. Chamomile tea composed of dried flower heads of German chamomile is a popular ingredient of herbal teas but adulteration is a major drawback in the production. Chromatographic techniques were compared for the evaluation of adulteration according to European Pharmacopoeia, which found that HPTLC was superior to the HPLC method for the detection of adulteration [172]. 

## 7. Safety Issues

German chamomile is used as a natural seasoning and flavoring agent and is generally recognized as safe (GRAS) for their utilization in food for individual consumption [173]. Additionally, they can be used as spices and other natural seasonings and flavoring agents that are GRAS for their potential use in feeds, animal drugs, and other related products [174]. The general detection of the safety and efficiency of the plant ingredients has led to their use in digestive-aid drug products [175]. The members of the Asteraceae family are known to cause sensitization, resulting in skin irritation and inflammation. The sesquiterpene lactones (SLs) have been thought to cause the allergenic potential of several Asteraceae species; allergy symptoms may include hay fever, asthma, eczema, or anaphylaxis [176]. Some individuals may develop clinical symptoms of vesicular hand eczema [177]. Another investigation was carried out to evaluate whether German chamomile tea can elicit possible systemic allergic dermatitis in patients allergic to sesquiterpene lactones, who were examined for the probable flare-up of healed-patch reactions to chamomile. The evaluated individuals were not found to have systemic allergic dermatitis or skin patch reactions [178]. 

## 8. Patents on Extraction and Medicinal Properties of German Chamomile

A study was conducted for patents on German chamomile in European Patent Society and World Intellectual Property Organization through databases viz., an Espacenet patent search, Canadian patents, Google patents, SciFinder, etc., dating from 1993 to 2015. The patents on extraction and medicinal properties of German chamomile are listed below.

### 8.1. Sub-Critical Water Extraction of Medicinal Plants

The invention presented an efficient method for obtaining extracts with useful pharmacological properties from German chamomile along with other medicinal plants without using an organic solvent. A method of extraction was developed using subcritical water to produce an extract with a similar composition as obtained from methanol or alcohol mixtures. The invention also included both oral and topical formulations to improve the bioavailability and efficacy of the therapeutic components of the extract [179].

### 8.2. Chamomile Oils Having a High Natural Spiro Ethers, and Process for Their Production

A process was developed for chamomile oil preparation containing at least one percent of natural spiro ethers by weight when subjected to steam or water distillation. The content of natural *cis*-spiro ether and *trans*-spiro ether was found to be at least 1 and 0.5 percent by weight. [180].

### 8.3. Chamomilla Extract for the Treatment of Hypertension

The process formulated a composition for treatment of hypertension, symptoms and diseases associated with hypertension. The formulated extract uses chamomile plants as the only active principle component [181].

### 8.4. Composition Containing Oils of Chamomile Flower and Black Cumin with Reduced Endotoxins

It has been found that a chamomile extract obtained by steam distillation can reduce DNA synthesis in human cancer cells and inhibit leukotrienes and IL-6 (interleukin 6) production. The volatile oil of chamomile has anti-inflammatory and anticancer properties alone or in combination with black cumin seed oil [182].

### 8.5. Acaricidal Properties of Flower of German Chamomile 

A safe and stable method of extraction was developed for ethanolic, hexane and chloroform fractions of German chamomile flower with acaricidal properties. Around an 80–90% mortality and 63–100% inhibition was observed in oviposition of Rhipicephalus (Boophilus) microplus using 8–10% of the extract. An efficacy of above 90%, 95%, and 95% was illustrated against R. (Boophilus) microplus using 4–5% of hexane, chloroform, and ethanolic extract, respectively. Ethanolic extract (60–70%) of chamomile flowers also revealed a mortality of deltamethrin-resistant ticks in the invention [183].

### 8.6. Oil Blend for Skin Treatment

The process cultivated a substantially anhydrous (no water phase at 70 degrees Fahrenheit) blend of oils comprising of German chamomile oil which is useful in the treatment of skin diseases. A blend of German chamomile oil with other oils, e.g., virgin coconut oil, olive oil, jojoba oil, tea tree oil, vitamin E oil, extra virgin calendula oil was found to be an effective treatment and method of prevention of skin ailments, such as diaper rash, eczema and in soothing the skin [184].

### 8.7. Hemostatic Dressing Comprising Extract of Chamomile and Nettle

The process involved the preparation of a hemostatic formulation of chamomile (*Chamomilla recutita*) flowers and the leaves of dioecious nettle (Urtica dioica). The addition of a biocompatible polymeric base (alignate) formulated an extract with broad spectrum hemostatic abilities, which had synchronized antiseptic and anti-inflammatory properties. The invention also provided methods and apparatuses useful in creative compositions to stop bleeding, as well as for use in a variety of hemostatic contexts. Hemostatic dressings comprise of a polymeric layer of formulation components that are applied to a fabric material [185].

## 9. Conclusions

From this review we conclude that German chamomile is a star herb which is extensively used in many homemade remedies, herbal drinks, condiments, and in the food and aroma industry, as has been the case since the classical period. Continuous research on its active constituents and pharmacological properties explored its importance in many medicinal formulations. Although it is a high value medicinal and aromatic herb, its cultivation is limited to kitchen gardens or small scale farmers. Farmers are not interested in its large scale cultivation due to a lack of good varieties, lack of low cost agrotechnologies for flower picking and the unsuitability of machine harvesting. Therefore, this crop required more research for its genetic improvement and varietal development for desired traits such as higher dry flower yield, synchronous flowering, similar flower pick length and uniformity in flowering flushes to facilitate and make machine harvesting efficient. The essential oil content and quality in the fresh and dry flower heads of German chamomile is highly variable and constitutes its most crucial trait, and depends on the origin of germplasm, ploidy level and extraction process. Hence, the essential oil content, quality and its extraction process also need to be improved to deliver benefits to farmers. The utilization of high throughput molecular markers such as SNPs for flowering and essential oil traits could hasten the genetic improvement through marker-assisted breeding in German chamomile. 

## Figures and Tables

**Table 1:** Major chemical components and their composition in essential oil of German chamomile.

Sr. No.	Major Components	Composition (%)	Population Origin	References
1	Chamazulene	11.0–21.0	Finland	[21]
		15.8–19.2	Germany	[17]
		0.7–15.3	European	[18]
		15.1	Iran	[22]
		8.4	Italy	[23]
		4.8–8.0	Iran	[20]
		1.0–6.6	Iran	[19]
		6.4	Bosnia and Herzegovina	[15]
		5.6	India	[24]
		4.7	Estonia	[25]
		4.2	Khorasan-Razavi, Iran	[26]
		2.6–3.3	Iran	[27]
		2.4	Iran	[16]
2	α-bisabolol	0.1–44.2	European	[18]
		23.9–44.2	Moldova, Russia and Czech Republic	[28,29]
		16.0	India	[24]
		5.6	Estonia	[25]
		5.0	Khorasan-Razavi, Iran	[26]
		2.4	Bosnia and Herzegovina	[15]
		1.7	Iran	[16]
3	α-bisabolol oxide A	57.81	Egypt	[30]
		3.1–56.0	European	[18]
		33.8–55.4	Iran	[20]
		7.3–55.3	Iran	[19]
		46.5	Egypt	[31]
		37.2–44.5	Iran	[27]
		43.8	Iran	[16]
		20.2–43.2	Moldova, Russia and Czech Republic	[28,29]
		39.4	Estonia	[25]
		36.5	India	[24]
		17.4–35.3	Germany	[17]
		21.5	Khorasan-Razavi, Iran	[26]
		17.14	Iran	[22]
		11.6–16.5	Brazil	[31]
		11.2	Italy	[23]
		7	Bosnia and Herzegovina	[15]
4	α-bisabolol oxide B	3.1–35.7	Iran	[19]
		15.5–35.6	Germany	[17]
		3.9–27.2	European	[18]
		9.9	Estonia	[25]
		8.6	India	[24]
		4.6–8.1	Iran	[20]
		3.7–7.1	Iran	[27]
		7	Khorasan-Razavi, Iran	[26]
		6.3	Bosnia and Herzegovina	[15]
		5.17	Iran	[22]
		3.8	Iran	[16]
5	α-bisabolone oxide A	8.3–39.9	Iran	[19]
		0.5–24.8	European	[18]
		11.7–16.5	Iran	[27]
		13.9	Estonia	[25]
		13.6	Iran	[16]
		10	Khorasan-Razavi, Iran	[26]
		4.9–9.1	Germany	[17]
		6.15	Iran	[22]
		3	Bosnia and Herzegovina	[15]
6	β-bisabolene	19.6	Iran	[16]
7	β-farnesene	29.8	Bosnia and Herzegovina	[15]
		2.0–19.7	Iran	[19]
		10.8–18.1	Iran	[20]
		13.3–15.4	Iran	[27]
		14	India	[24]
		2.3–6.6	European	[18]
		5.2	Khorasan-Razavi, Iran	[26]
		2.7–3.9	Germany	[17]
8	α-farnesene	9.3	Bosnia and Herzegovina	[15]
		3.1	Iran	[16]
9	*cis*-en-yn-dicycloether	8.8–26.1	European	[18]
		9.7–24.2	Iran	[19]
		11.5	Estonia	[25]
		6.2	Iran	[22]
10	Spathulenol	9.4	Khorasan-Razavi, Iran	[26]
		3.4	Iran	[16]
11	Germacrene D	6.2	Bosnia and Herzegovina	[15]
		3.0	Iran	[22]
12	Occidol acetate	7.2–13.4	Iran	[20]
13	Isobornyl isobutyrate	11.1–14.0	Germany	[17]
14	*Z*-spiroether	5.6–9.9	Iran	[27]
		5.1	Bosnia and Herzegovina	[15]
15	*n*-Octanal	6.0	Iran	[22]

**Table 2:** Properties and their utilizations of German chamomile.

Sr. No.	Properties	Utilizations	References
1	Organoleptic	Flavor, taste and color of food	[52]
2	Anti-allergic	Effective against allergic reactions	[53,54,55]
3	Anti-spasmodic	Abdominal pain, to relax intestinal muscles and irritation	[56,57]
4	Anxiolytic	Effective against anxiety	[58,59,60,61]
5	Anti-inflammatory	Penetrate deep in skin and reduce redness, swelling, pain and eye irritation	[26,62,63,64]
6	Anti-microbial	To inhibit growth of bacteria (Gram-positive and Gram-negative) and fungi	[26,65,66,67,68,69]
7	Neuroprotective	Helpful in recovery of neuro disorders	[55]
8	Sedative	Induce sleep, sedation and calming effects	[70,71]
9	Anti-oxidant	Rich source of antioxidants	[55,72]
10	Anti-depressive	Stimulant to relax the muscles and effective against depression	[72,73]
11	Anti-cancer	Control over cancerous cells	[55,74]
12	Hepatoprotective	To recover liver damage	[55,72,75]
13	Anti-diarrheal	In treatment of children’s colic and diarrhea	[72,76,77]
14	Gastrointestinal cure	To sooth bowl movement and flatulence	[72,78]
15	Healing	Wound healing	[79,80,81,82,83]
16	Anti-viral	Relief in common cold and inhibit Poliovirus	[66,84]
17	Anti-ulcer	Mouth ulcers, intestinal irritations and ulcers	[85,86]

## Data Availability

Not applicable.
